# The City of Johannesburg Metropolitan Municipality as a Microcosm of Fruit and Vegetable Waste Governance Failure in Low- and Middle-Income Countries: Evidence from Formal and Informal Retailers

**DOI:** 10.3390/ijerph23040456

**Published:** 2026-04-03

**Authors:** Mpinane Flory Senekane, Cavin Omphemetse Moreetsi

**Affiliations:** Department of Environmental Health, Faculty of Health Sciences, University of Johannesburg, Doornfontein, P.O. Box 524, Johannesburg 2006, South Africa

**Keywords:** fruit and vegetable waste, low- and middle-income countries, urban food systems, awareness, practices

## Abstract

**Highlights:**

**Public health relevance—How does this work relate to a public health issue?**

- Poorly managed Fruit and Vegetable Waste (FVW) is linked to imminent negative environmental and public health effects, which may subsequently impact public health outcomes in urban areas like the City of Johannesburg Metropolitan Municipality (COJMM).
- This study addressed awareness and management practices of FVW among formal and informal retailers, providing evidence to support improved FVW management interventions and policies that enhance sustainability and protect public health.

**Public health significance—Why is this work of significance to public health?**

- The study addresses a gap in urban FVW governance in low- and middle-income countries (LMIC) by displaying how institutional and operational shortcomings in COJMM affect FVW management outcomes, contributing to the body of literature addressing public health governance.
- The findings provide practical evidence for municipalities to improve enforcement, strengthen regulatory frameworks, and design interventions that enhance effective FVW management.

**Public health implications—What are the key implications or messages for practitioners, policy makers, and/or researchers in public health?**

- Effective retail FVW management requires stronger governance systems, particularly in municipalities such as the City of Johannesburg Metropolitan Municipality and similar urban LMIC settings.
- Practitioners, policy makers, and researchers should prioritize integrated, context-specific interventions that address infrastructural, institutional, and operational challenges to FVW management.

**Abstract:**

FVW has become a major sustainability concern due to rapid urbanization and rising demand for fresh produce in LMIC. This study investigates FVW governance by assessing awareness levels and FVW management practices among formal and informal fruit and vegetable retailers in Region F of the COJMM. Quantitative, descriptive design was employed. Data were collected using questionnaires that assessed demographic details, awareness of FVW, and current FVW management practices. The findings from 43 formal and 118 informal retailers revealed fragmented governance across the retail sectors. 54.2% of informal retailers reported being unaware of municipal waste management by-laws, and 83.9% indicated that disposal was the only effective waste management method. Formal retailers showed greater awareness of the impacts of poor FVW management (83.7% agreed or strongly agreed), yet 65.1% still relied primarily on disposal practices. The findings show limited awareness of municipal waste governance tools and reliance on disposal, with minimal FVW valorization. Chi-square results showed that age, gender, and education significantly influence awareness among informal retailers, while no significant differences were found among formal retailers. The study concludes that unsustainable FVW management is driven by structural governance limitations, highlighting the need for inclusive approaches to improve urban FVW governance in LMIC.

## 1. Introduction

Urban food systems in low- and middle-income countries (LMIC) are experiencing a rapid transformation due to accelerated urbanization, population growth, and increased demand for fresh produce [1]. Among the diverse categories of food waste generated within cities, FVW accounts for the largest proportion by volume and weight. This is due to their high perishability nature, as they can be vulnerable to high temperature exposure, mechanical damage, and poor storage conditions [2]. Therefore, FVW represents a recognizable and persistent challenge within urban contexts, necessitating proper governance systems that enhance sustainability.

In LMIC cities, FVW is predominantly generated during the distribution and retail phases of the supply chain [3]. The urban fruit and vegetables (FV) retail sector, including both formal and informal retail spaces, is continuously strained and forced to operate under unfavorable conditions characterized by limited cold-chain infrastructure, fluctuating daily demand, and constrained storage capacity [4]. These structural limitations greatly exacerbate the likelihood of FVW spoilage, leading to a rapid accumulation of FVW.

Rapid urbanization has further exacerbated this challenge. The increase in urban populations has widened dependence on the informal retail sector, which poses crucial challenges in terms of food systems’ sustainability, including urban food waste [5]. Informal retailers often operate in open-air environments with inadequate infrastructural support, a lack of access to waste collection services, and restricted inclusion in municipal planning. Informal retailers are a vital component of urban food security; therefore, their exclusion from formal waste governance frameworks contributes to ineffective FVW management within retail environments [6].

The City of Johannesburg Metropolitan Municipality (COJMM) represents a crucial node within South Africa’s urban food system. As South Africa’s economic nucleus, the city is characterized by an extensive formal and informal FV retail activity, including large-scale formal wholesale markets, e.g., Joburg Market, and widespread informal FV retailers [7]. These retailers play a vital role in ensuring food access for a diverse urban population; however, they also generate substantial volumes of FVW daily. The management of FVW within COJMM, therefore, constitutes a significant urban environmental and governance concern. In this study, governance refers to the operations, regulations, institutional coordination, and stakeholder engagement that guide retailers’ effective management of FVW. In many retail environments, there has been evidence of poor waste segregation, informal dumping, or temporary accumulation near stalls and markets [8,9,10]. Inadequate separation at source and insufficient infrastructure for organic waste diversion further exacerbate the problem. The COJMM appointed Pikitup as its primary waste management service provider, with two primary objectives: achieving “Zero waste sent to landfills by 2022” and “promote recycling”. COJMM generates over 1.4 million tons of municipal waste per year, of which only 13% is recycled. This provides evidence that Pikitup has not been able to meet the set objectives, signaling a significant waste management failure in the COJMM [11]. These reflect not only operational constraints but also broader weaknesses in urban food waste governance.

Within this context, the COJMM can be recognized as a microcosm of urban FVW governance failure in LMIC. The city exhibits many structural characteristics similar to other LMIC urban environments, including high levels of socio-economic inequality, dependency on informal food systems, infrastructural disparities, and a lack of effective municipal waste management systems [5]. The challenges in waste governance observed in the COJMM, therefore, mirror those experienced in many LMIC cities. This allows the COJMM to be used as a representative case through which broader systemic failures in urban waste governance within LMIC can be examined.

Poor management of FVW poses a critical environmental threat due to its high organic content. When FVW is disposed of in landfills, anaerobic decomposition releases greenhouse gases such as methane (CH_4_) and other harmful emissions, including ammonia and volatile organic compounds (VOCs), which lead to climate change and air pollution [12]. The emissions from decomposed FVWs directly impact human health by irritating the respiratory system and affecting multiple organ systems, depending on the gaseous concentrations [13]. Landfill leachate and gases produced from decomposed organic waste can lead to contamination of soil and water resources. This leads to a degradation of the ecosystem and increases the risk of waterborne pollutants entering surface and groundwater [14]. Additionally, FVWs create favorable conditions for vectors, such as flies and rodents, thereby increasing the likelihood of pathogen spread and vector-borne diseases in nearby communities [12]. This underscores the public health importance of effective FVW governance within urban settings.

In the COJMM, these environmental and public health risks are evident, as the area is densely populated around the Central Business District (CBD), where large volumes of FV are traded daily through both formal and informal channels. FVW often accumulates in trading spaces due to inadequate waste segregation, limited storage infrastructure, and inconsistent waste collection services. The accumulation of FVW in open environments can attract pests, generate unpleasant odors, and contribute to unsanitary conditions that pose health risks to surrounding communities [15]. These challenges illustrate how broader FVW governance issues manifest within the urban context of COJMM.

Despite the significance of FVW within urban food systems, existing research has predominantly focused on street vendors’ food waste or general organic waste management; limited studies have examined FVW specifically at the retail level. For example, ref. [16] investigated FVW management behavior among retailers in Kumasi, Ghana, providing insights into FVW management. However, to our knowledge, no studies have compared formal and informal FVW governance within African metropolitan contexts. This study, therefore, represents the first effort to assess and compare formal and informal retail sectors in terms of FVW awareness and management practices within an African city.

Consistent with these findings, this study aims to investigate FVW governance within the COJMM region F, focusing on both formal and informal retailers by assessing their awareness levels regarding FVW and examining their current FVW management practices. This will aid in addressing the research questions for this study, which are:“ How is the governance of FVW in COJMM? What is the awareness level of retailers in Region F of the COJMM on FVW? What are the current practices aimed at effectively managing FVW by retailers in the COJMM Region F? The contribution of this study seeks to add to the body of science, enhancing understanding of urban FVW governance failures in LMIC contexts and informing the development of inclusive and sustainable urban waste management strategies.

## 2. Materials and Methods

### 2.1. Description of Study Area

**Figure 1:**
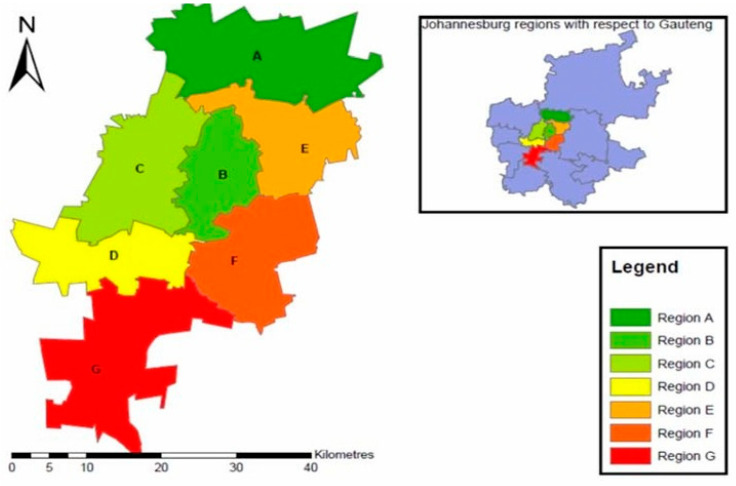
City of Johannesburg Regions [18].

The study was conducted in the City of Johannesburg Metropolitan Municipality (COJMM) Region F, Gauteng Province. See Figure 1. Region F represents the inner city of the City of Johannesburg Metropolitan Municipality and serves as the historic and economic core of the metropolitan area, with an estimated population of approximately 658,000, representing about 13.3% of the total population of Johannesburg. The population is predominantly young and economically active, with 44% of residents aged 25–44.

Economically, Region F is the highest GDP-generating region within Johannesburg and employs the largest share of workers in the metropolitan municipality. Estimates suggest that approximately 10,000 street traders operate within the inner city, generating around R4.2 billion annually [17].

### 2.2. Study Design

The study employed a quantitative descriptive design, utilizing a non-experimental survey to investigate FVW governance within the COJMM region, focusing on both formal and informal retailers.

### 2.3. Study Population

In this study, the population refers to formal franchise supermarkets (formal retailers) and informal fruit and vegetable retailers (informal retailers). The study’s targeted population comprised 61 formal and 1000 informal retailers. The total number of formal franchise supermarkets within Region F was 61. The number of informal retailers exclusively engaged in the sale of FV cannot be definitively confirmed due to the persistent fluctuations in their numbers. However, based on advice received from an expert who regularly engages with informal FV retailers, an estimate of approximately 1000 retailers was provided.

### 2.4. Sampling

A purposive sampling approach was employed to identify retailers actively engaged in the sale and handling of FV within Region F of the COJMM. This method was considered appropriate given the heterogeneous and poorly documented nature of the target population, particularly in the informal sector, where many operators are unregistered, mobile, and absent from official municipal records. As a result, the application of probability-based sampling techniques was not practical. The study deliberately targeted two categories of retailers with high levels of exposure to FVW generation: informal traders operating in public market spaces and formal franchise supermarkets. This sampling strategy facilitated the inclusion of participants with substantial practical experience in FVWs management, thereby strengthening the validity and contextual relevance of the findings.

### 2.5. Sample Size Estimation

Using Epi Info version 7, the following parameters were used to determine the sample size: a 90% confidence level (CL) and a ±7% margin of error. A 90% confidence level was selected to balance reliability with the practical constraints of field-based data collection, particularly when engaging informal retailers who are often mobile and difficult to reach during working hours. The estimated sample sizes in the study were 121 and 43 for informal and formal retailers, respectively. The sample achieved for formal retailers was 43, and 118 for informal retailers

### 2.6. Data Collection and Pilot Study

Prior to the main data collection, a pilot study was undertaken in Region F of the COJMM to assess the appropriateness of the research instrument and refine the overall data collection procedure. The pilot involved a small sample comprising ten informal fruit and vegetable retailers and five formal retailers. Participants were requested to complete the same questionnaire used in the main study, corresponding to their respective retail categories.

The ethical protocols applied in the main study were also implemented during the pilot phase. Participants were duly informed about the purpose of the study, provided with the study’s information letter, and required to give written consent prior to participation. The pilot exercise was critical for detecting ambiguous or poorly structured items and allowed for necessary revisions to enhance the comprehensiveness and clarity of the questionnaire. Individuals who took part in the pilot were subsequently excluded from the main study to avoid potential bias.

Data were gathered through a structured quantitative questionnaire administered by the researchers to both formal and informal retailers. The instrument comprised three sections: the first captured respondents’ demographic information, the second evaluated levels of awareness among retailers, and the third examined existing practices related to FVW management. For this study, awareness is defined as retailers’ knowledge and understanding of FVW management and related subjects, for example, waste management by-laws or the impacts of poor FVW management [19]. FVW management practices are defined as the daily actions and behaviors retailers employ to manage FVW, for example, segregation, repurposing, etc. [20]. To maintain participant anonymity throughout the data collection process, respondents were identified using coded labels rather than personal identifiers. Formal franchise supermarkets were designated with the code “A”, while informal retailers were assigned the code “B”. The questionnaire used in this study was developed by the researchers based on the research question and objectives. The questionnaire was structured using a Likert-scale format. This format was selected because retail environments are often fast-paced, with retailers primarily focused on serving customers. Consequently, the Likert-scale design enabled the researchers to collect data efficiently, as such questions are simple, quick to complete, and suitable for respondents with limited time. Other questions used to assess formal retailers’ practices were in multiple-choice and dichotomous formats. The researcher utilized a handheld device for data collection. The researcher captured respondents’ responses in a Google Forms sheet while asking the questions. Data were collected over 3 months (January 2025 to March 2025). Questionnaires were distributed to 1 overseer per market (a manager in a formal retail store and the person found selling in an informal market). Participants were approached at their workplaces during standard business hours, Monday to Friday. In instances where immediate questionnaire administration was not feasible, follow-up appointments were scheduled at times that suited the respondents’ availability.

### 2.7. Data Analysis

The data collected from the questionnaires were analyzed using IBM SPSS Statistics software, version 30.0. Data extracted from Google Forms was transformed into an Excel spreadsheet and subsequently imported into SPSS. The data was initially cleaned to remove missing values, typographical errors, duplicates, and anomalies. Data cleaning is a crucial step in the analysis process to ensure quality, as it helps eliminate inaccurate data that could lead to unreliable results [21]. Descriptive analysis was employed to present the study findings through frequency distributions and percentages. The results are displayed in tables and graphs. To address Objective 1, Pearson’s Chi-square tests were applied to examine associations between selected demographic variables, namely age, gender, and educational attainment, and levels of awareness. Statistical significance was determined at *p* < 0.05

#### Inferential Analysis

A. Description

As indicated in Table 1, Pearson’s Chi-square test was utilised to assess the study’s hypotheses. These analyses were conducted across both datasets, representing informal and formal FV retailers. The hypotheses were developed based on the awareness construct, as the questions used to measure this construct were consistent across both formal and informal FV retailers. This will enable the researcher to compare the differences across these sectors regarding awareness. Although the questions used to assess the awareness construct were identical to maintain consistency and comparability, the hypotheses were conceptually framed to account for differences in their operational contexts. Specifically, it was expected that the relationship between demographic factors and awareness could differ between the two groups, with informal retailers being more affected by individual traits, while formal retailers’ awareness might be influenced by structured training and institutional procedures.

The following hypotheses are tested:

**H1a.**  *There is no statistically significant difference in awareness of FVW among retailers of different age groups in Region F of the COJMM.*

**H1b.**  *There is a statistically significant difference in awareness of FVW among retailers of different age groups in Region F of the COJMM.*

**H2a.**  *There is no statistically significant difference in awareness of FVW among retailers of different genders in Region F of the COJMM.*

**H2b.**  *There is a statistically significant difference in awareness of FVW among retailers of different genders in Region F of the COJMM.*

**H3a.**  *There is no statistically significant difference in awareness of FVW among retailers of different educational levels in Region F of the COJMM.*

**H3b.**  *There is a statistically significant difference in awareness of FVW among retailers of different educational levels in Region F of the COJMM.*

### 2.8. Ethical Clearance

The study was approved by the University of Johannesburg’s Research Ethics Committees (REC-3201–2024). Permission was obtained from the City of Johannesburg Metropolitan Municipality’s Department of Corporate & Shared Services in the office of the Group Head: Group Human Capital Management. Permission was also obtained from the Johannesburg Property Company (JPC). The study was conducted in accordance with the local legislation and institutional requirements. Prior to the commencement of the study, respondents were informed of its purpose, nature, and extent, as well as how data would be collected. This was done through a study information letter. Respondents were provided with all the information throughout the study. Respondents were informed about the researcher’s role. Respondents were also asked to complete a consent form, which allowed the researcher to include them in the study.

## 3. Results

### 3.1. Section 1 (Informal Retailers)

#### 3.1.1. Socio-Demographic Characteristics of Informal Retailers

A total of 118 informal retailers participated in the study. As presented in Table 2, the sample comprised 77 males (65.3%) and 41 females (34.7%). The largest proportion of respondents fell within the 30–49 age range (67.8%), whereas the smallest group was aged 70–89 (0.8%). Regarding educational attainment, most participants had completed primary education (39%), while only a small fraction had tertiary qualifications (3.4%)

#### 3.1.2. Informal Retailers’ Awareness Level

This subsection examined the awareness levels of informal retailers, measured using a Likert scale, with results summarized in Table 3.

Participants were asked about their awareness of the consequences of improper FVW management. Most respondents (39.8%) indicated that they were aware, while 33.1% disagreed. A very small proportion (1.7%) strongly agreed that they fully understood the impacts of FVW mismanagement. When questioned on whether FVW represents a global issue, 31.4% disagreed, 28.8% agreed, and 22% strongly disagreed, resulting in a clear predominance of disagreement; no participants strongly agreed.

Regarding awareness of the COJMM’s waste management by-laws, the majority (54.2%) indicated disagreement, 33.1% were neutral, and only a small fraction expressed agreement. When asked if disposing of FVW is the sole effective method of waste management, a large majority (83.9%) agreed, 11% were neutral, and none strongly disagreed.

#### 3.1.3. Informal Retailers’ FVW Practices

This subsection explored the Informal retailers’ FVW practices using a Likert scale, as seen in Table 4.

Respondents were asked whether they were inducted on ways they can use to minimize FVW. A large proportion (81.4%) disagreed, followed by 13.5% who were neutral. As only 1.7% strongly agreed, while none (0%) of the respondents agreed. When asked if they store FV in suitable conditions. Most (44.9%) disagreed, followed by 32.2% of respondents who agreed,

Respondents were asked if they mix FVW with other waste types when disposing. A substantial number (42.4%) agreed with the statement; however, 35.6% of respondents still disagreed. The respondents were asked if they were inducted on the proper disposal of FVW. Most respondents disagreed (44.9%), followed by 28.8% who agreed. This mixed response is corroborated by their responses in the “*mixing of waste*” question.

### 3.2. Section 2 (Formal Traders)

#### 3.2.1. Socio-Demographic Characteristics of Formal Retailers

As shown in Table 5, the study included 43 formal retailers, comprising 25 females (58.1%) and 18 males (41.9%). The largest age group was 18–29 years (60.5%), while the smallest was 50–65 years, with only one participant (2.3%). Most respondents had completed secondary education (60.5%), and none had either no formal education or only primary-level schooling. A notable portion (39.5%) had attained tertiary education.

#### 3.2.2. Formal Retailers’ Awareness Level

This subsection examined the awareness levels of formal retailers, measured using a Likert scale, with results summarized in Table 6.

The majority of respondents indicated awareness of the consequences of poor fruit and vegetable waste (FVW) management, with 46.5% strongly agreeing and 37.2% agreeing. When asked whether FVW is a global concern, responses were more varied: 25.6% disagreed, 23.3% agreed, and 20.9% strongly disagreed, indicating an overall tendency toward disagreement.

Concerning awareness of the COJMM’s waste management by-laws, over half of the respondents (54%) reported a neutral stance. This was followed by 27.5% who indicated agreement, while a smaller segment (11.6%) disagreed, suggesting varied levels of knowledge regarding the regulations. Regarding the perception that disposal is the sole effective method for managing fruit and vegetable waste, most respondents agreed (65.1%), with an additional 32.6% strongly agreeing.

#### 3.2.3. Formal Retailers’ FVW Practices

The researcher made some amendments to the questions used to determine the formal retailers’ FVW management practices. This was because some questions were not relevant to an informal setting; however, they can be addressed in the formal retail sector.

**Figure 2:**
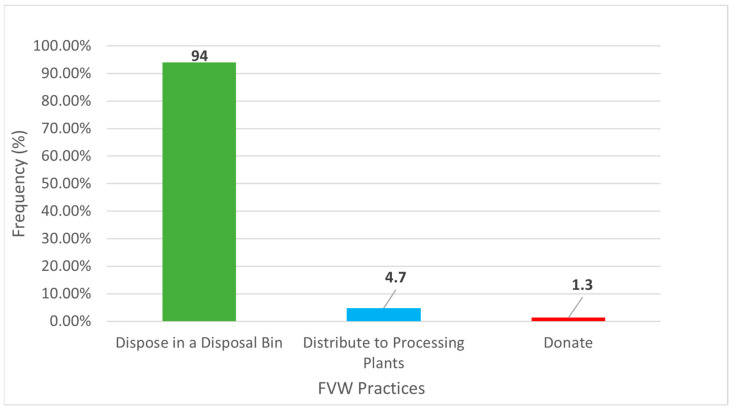
How does the store manage of unconsumable fruit and vegetables?

As seen in Figure 2, the common practice among formal retailers for disposing of FVW is to use a disposal bin (94%), followed by sending it to processing plants (4.7%), and then donating it (1.3%). This is also supported by the question “*Disposal of fruit and vegetable waste as the only effective waste management method*,” which confirms that disposal is the standard practice in the retail sector when handling FVW.

When asked whether FVW should be mixed with other waste types, the majority of respondents selected a neutral response (72.1%), as seen in Table 7. Formal retailers’ FVW practices. This was followed by respondents who disagreed (9.2%) and strongly disagreed (14%), indicating varied positions regarding FVW segregation practices. A strong consensus was observed regarding storage practices, with most respondents (55.8%) strongly agreeing or (44.2%) agreeing that FV were stored under suitable conditions. Similarly, most respondents reported having received induction on ways to minimize FVW, with 69.8% agreeing and 27.9% strongly agreeing. The researchers further administered two questions to formal retailers to corroborate responses to the question “*induction on ways to FVW.*”

**Figure 3:**
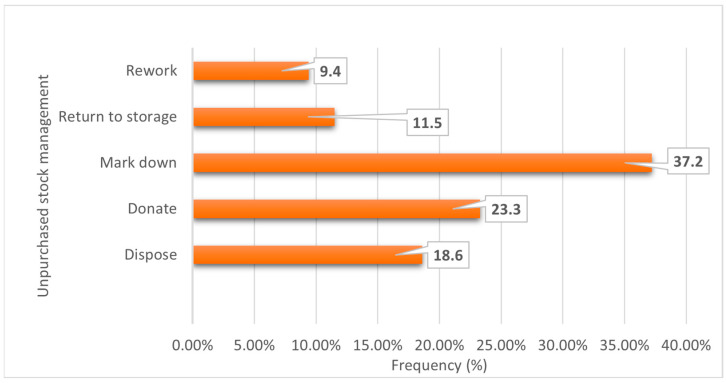
How does the store manage unpurchased fruit and vegetable stock?

Figure 3 illustrates variability in how stores manage unsold stock. The most common practice was marking down products (37.2%), followed by donation (23.3%), while a smaller proportion returned stock to storage (11.5%). Disposal was reported by 18.6% of respondents.

**Figure 4:**
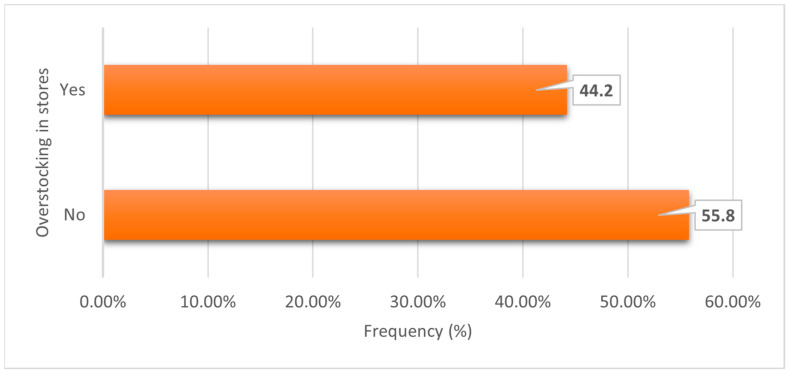
Is there a tendency to overstock fruit and vegetables in the store?

Figure 4 shows whether there was a tendency to store more stock than required; over half (55.8%) reported stocking only what was needed, while 44.2% indicated overstocking.

### 3.3. Chi-Square Test Results: Informal FV Retailers

The variables included in the Chi-square test comprised gender, age, educational levels, and items measuring the awareness construct. A summary of the Chi-square test results is provided in Table 8.

The results of the chi-square test are categorized based on age, gender, and educational level. Regarding age, most questions (3 out of 4) showed a significant difference in awareness levels about FVW (H1b), rejecting the null hypothesis (H1a). For gender, all results indicated a significant difference in awareness levels between male and female informal FV retailers (H2b); therefore, the null hypothesis is rejected. Regarding educational level, most questions (3 out of 4) also showed a significant difference in FVW awareness by retailers’ educational background in the COJMM, Region F, leading to the rejection of the null hypothesis (H3a). These findings suggest that age, gender, and educational level among informal retailers influence their awareness levels of FVWs.

### 3.4. Chi-Square Test Results: Formal FV Retailers

The variables included in the Chi-square test comprised gender, age, educational attainment, and items measuring the awareness construct. A summary of the Chi-square test results is provided in Table 9.

The chi-square test results were also analyzed by gender, age, and educational level. Regarding age, 1 of 4 questions showed a significant difference in the level of awareness of FVW across age groups of formal FV retailers in the COJMM, Region F. Most of the questions’ *p*-values are above 0.05, which means in the broader context, age is not statistically significant in the level of awareness regarding FVW among formal FV retailers. The null hypothesis (H2a) is accepted for the gender level across all questions, as the *p*-values were all higher than 0.05. For educational level, all questions showed no significant difference in awareness level, thereby accepting the null hypothesis (H3a). Therefore, these results prove that age, gender, and the educational level of the formal FV retailers do not contribute to the level of awareness they possess regarding FVWs.

## 4. Discussion

### 4.1. Demographics of Respondents

The demographic profile observed in this study reflects patterns commonly observed in LMIC urban food systems. Table 2 in Section 3 shows the dominance of male participants in the informal sector, mirroring gendered labor dynamics reported in studies conducted in South Africa and Bangladesh, where informal street trading is often male-dominated [22,23]. Higher female participation in the formal retail sector is evidence that the formal retail sector offers more structured and accessible employment opportunities. A similar demographic profile was observed in a study conducted in Ghana, whereby females dominated the formal work force [24]. These gendered patterns should be viewed within the broader context of informal sector livelihoods and the obstacles to formalization in LMICs. Participation in informal retail is frequently constrained by limited access to capital, regulatory challenges, and complex business registration procedures, all of which more strongly hinder entry into the formal economy. A substantial proportion of the global workforce, exceeding 60%, derives its livelihood from the informal economy. Although informality is a universal phenomenon observed across countries at varying levels of socio-economic development, it is particularly prevalent in LMIC. An estimated 2 billion men and women are engaged in informal economic activities due to non-decent working conditions. Evidence shows that most people enter the informal economy not by choice but because of a lack of opportunities in the formal economy and the absence of other means of livelihood [25,26]. This suggests that these structural barriers sustain the prevalence of informal trade and shape the demographics of those involved in these sectors.

The age distribution of respondents further reflects retail food trade as an important tool in the livelihood of populations in LMIC cities. Informal retailers were mostly middle-aged, relying largely on experiential knowledge, while formal retailers were generally younger and more likely to be exposed to better educational attainments. Previous studies suggest that younger, formally employed workers have a higher degree of environmental concern and environmentally responsible behavior [27]. This suggests that formal retailers may be more receptive to structured waste management initiatives. However, such exposure does not necessarily guarantee the adoption of sustainable practices in the absence of supportive governance frameworks.

Educational disparities are observed between informal and formal retailers (as seen in Table 2 and Table 5), with informal retailers mostly possessing primary or no formal education, while formal retailers commonly possess secondary or tertiary qualifications. However, the prevalence of disposal-dominated practices among better-educated formal retailers suggests that governance failures are primarily structural rather than individual.

### 4.2. Fragmentation in FVW Governance Across Retail Systems

The findings show that FVW governance in COJMM is characterized by fragmentation across formal and informal retail systems, a trait commonly reported in LMIC urban contexts [28]. In this study, governance refers to the operations, regulations, institutional coordination, and stakeholder engagement that guide retailers’ effective management of FVW. Informal retailers are largely disconnected from municipal waste governance mechanisms, while formal retailers operate within structured corporate and regulatory environments dictated at head office levels. This dichotomy mirrors literature from other LMIC cities where waste governance frameworks tend to favor formal actors, resulting in uneven implementation and ineffective waste management systems [5].

In this study, governance refers to the operations, regulations, institutional coordination, and stakeholder engagement that guide retailers’ effective management of FVW, whereas management refers to the operational practices they implement. The analysis is informed by the Theory of Planned Behavior (TPB), which highlights how institutional structures, enforcement, and stakeholder inclusion can drive compliance behaviors [29].

The reported lack of awareness of COJMM waste by-laws among informal retailers, concurrently with mixed awareness dominated by uncertainty among formal retailers, suggests that regulatory instruments alone are insufficient to ensure effective governance. On-the-ground enforcement by authorities is required to ensure a consistent awareness and implementation of these bylaws by retailers. According to a study conducted in Uganda, Kampala, there was a display of how poor communication, limited enforcement capacity, and the exclusion of informal actors undermine urban waste governance outcomes, even where policy frameworks formally exist [30].

Similar governance challenges have been reported in other LMIC cities, including Dhaka (Bangladesh), Nairobi (Kenya), and Accra (Ghana), where informal retailers are frequently excluded from municipal waste systems, enforcement is weak, and infrastructural support for waste separation is limited [5,31,32]. These comparative examples support the argument that the fragmentation and systemic gaps observed in COJMM are not unique but reflect broader LMIC urban FVW governance patterns.

A significant number of “Neutral” responses to questions about by-laws and waste mixing suggest they reflect uncertainty instead of true indecision. These neutral answers could stem from unclear questionnaire wording, a limited grasp of municipal rules, hesitance to acknowledge non-compliance, or survey fatigue. Such neutrality underscores the need for targeted training and more transparent communication from municipal authorities to reduce uncertainty and enhance adherence to FVW governance protocols.

### 4.3. Prevalence of Disposal-Oriented Practices

Regardless of moderate to high awareness of the impacts of poor FVW management, especially among formal retailers, disposal remains the prevalent FVW management approach across both sectors. This awareness–practice gap has been widely highlighted in LMIC food systems research, where structural and institutional constraints limit the adoption of sustainable practices regardless of individual awareness levels [16].

This observed disposal-oriented practice suggests that COJMM’s challenges are not primarily behavioral but systemic. A study conducted in Nigeria reported that limited access to waste valorization infrastructure, weak incentives, and cost-driven operational priorities constrain the ability to move beyond disposal, reinforcing unsustainable waste management pathways [33].

The strong perception among respondents that disposal remains the only effective method for managing FVW reflects a broader governance norm in COJMM, where waste systems remain primarily reliant on collection and landfill disposal. This finding aligns with global assessments indicating that separation and organic waste valorization are less recognized in many LMIC urban settings [34].

The underutilization of donation and repurpose methods among formal retailers when managing FVW further supports evidence that such alternatives are constrained by inadequate institutional support, logistical barriers, and limited coordination between retailers and municipalities. COJMM experience, therefore, reflects structural barriers that are common across LMIC urban food waste systems rather than isolated local failures. In interpreting responses on mixing FVW with other waste types, the high proportion of neutral responses (72%) suggests that respondents may have been uncertain about appropriate practices rather than indifferent. This reinforces the observation that awareness alone is insufficient and that clarity of regulations, improved guidance, and practical training are needed to translate knowledge into action.

### 4.4. Informal Retailers and Structural Exclusion from Waste Governance

The evident gaps in induction and training on FVW minimization practices among informal retailers underscore their ongoing exclusion from formal waste governance structures. Similar patterns have been reported in Kenya and Bangladesh, where informal retailers operate in regulatory grey zones and have limited technical or institutional support for sustainable waste management [5].

Significantly, the observed practices within the informal sector should be interpreted within the context of constrained operating environments. Previous studies highlight that a lack of storage infrastructure, limited access to effective waste management services, and inconsistent municipal support significantly affect waste generation and disposal behaviors in the informal retail sector [34]. These findings imply that failure in governance does not emerge from unwillingness to comply, but from structural neglect within urban waste management systems.

### 4.5. Persistence of Inefficiencies in Formal Retail Practices

Although formal retailers demonstrated stronger storage practices and reported higher exposure to FVW minimization training, the continued reliance on disposal and the existence of overstocking practices in a substantial proportion of outlets highlight persistent inefficiencies. Monitoring and support from relevant authorities can translate formal retailers’ strong practices and inductions into actions that align with the circular economy principles. A study conducted in Ghana indicated a lack of effective monitoring and supervision from authorities as a driver of ineffective management systems [16].

The coexistence of internal FVW minimization policies with disposal-dominated practice reinforces the argument that governance challenges extend beyond individual retailers. This poor governance thereby influences the adoption of unsustainable practices by formal retailers. This supports the argument from studies suggesting that a lack of municipal involvement in store policy implementation or the absence of relevant national policies and regulations is a driver of limited adoption of circular economy practices among formal retailers [35].

### 4.6. COJMM as a Microcosm of Broader LMIC Urban FVW Governance Failure

Collectively, these findings display that FVW governance challenges in COJMM reflect broader systemic failures commonly observed in LMIC cities. The concurrent existence of policy frameworks, partial awareness, and persistent unsustainable practices across both formal and informal retail sectors underscores the limitations of existing governance systems. The researchers argue that successful FVW governance within a municipality cannot be achieved solely through the existence of policies and supporting systems implemented by municipal authorities. High levels of awareness and the adoption of strong, sustainable practices among retailers are also necessary to ensure an effective FVW governance system.

The coexistence of formal policies, partial awareness, and persistent unsustainable practices among retailers in COJMM indicates systemic governance failure, consistent with findings in other LMIC urban contexts. Evidence from Kampala (Uganda), Dhaka (Bangladesh), Nairobi (Kenya), and Accra (Ghana) shows that weak enforcement, exclusion of informal actors, and limited infrastructure similarly hinder effective urban FVW governance. By placing COJMM within this comparative evidence, we argue that the challenges observed are systemic rather than isolated local issues.

Previous studies on LMIC urban waste governance accentuate the need for integrated, inclusive, and capacity-sensitive governance approaches that bridge formal–informal disconnect, strengthen municipal–retailer engagement, and expand infrastructure for FVW separation and valorization [35,36,37]. The evidence from COJMM supports this body of literature, situating the city as a representative case through which broader urban FVW governance failures in LMIC can be examined. While COJMM shows patterns typical of LMIC urban FVW governance, it also displays context-specific features, such as relatively well-funded municipal waste infrastructure and organized formal retail chains. These aspects add important contextual details but do not reduce COJMM’s value as a representative case for studying broader governance trends in LMIC cities.

### 4.7. Role of Demographics and Inferential Analysis

The socio-demographic characteristics of retailers in Region F exhibit considerable diversity, providing important context for understanding variations in awareness. Notably, differences between informal and formal retailers extend beyond operational practices to include the composition and attributes of their respective workforces.

#### 4.7.1. Informal Retailers Awareness Gap

Limited formal education appears to be associated with substantial gaps in awareness, particularly regarding COJMM waste management by-laws and the global significance of FVW. Inferential analysis (Table 8) indicates that educational attainment has a significant effect on awareness levels among retailers, leading to the rejection of H01c. These findings are consistent with international studies showing that limited educational attainment can impede comprehension of complex environmental policies, awareness of global environmental issues, adoption of responsible practices, and understanding of circular economy principles [27]. In the absence of targeted educational initiatives designed to accommodate varying literacy levels, these gaps in awareness are likely to persist.

#### 4.7.2. Formal Retailers Consistent Awareness

Inferential analysis of formal retailers (Table 9) revealed that demographic variables—including age, gender, and educational attainment—did not significantly influence awareness levels. This finding suggests that corporate training programs and standardized operating procedures effectively promote a consistent level of awareness among employees, irrespective of their individual demographic characteristics.

## 5. Limitations

The researchers recognize several limitations that may have influenced the study’s findings. Consequently, further research is necessary to address these constraints and contribute to the broader body of literature, enhancing scientific understanding of the topic. The specific limitations were identified both in the literature review and throughout the data collection process.

### 5.1. Limitations in the Literature

A comprehensive review of the literature is fundamental to any research. In this study, a limited number of sources were available for the specific areas under investigation, thereby constraining the scope of existing knowledge. The researchers viewed this limitation as an opportunity to identify gaps in prior studies and emphasize directions for future research.

### 5.2. Limitations During Data Collection

- Time

For informal retailers, the time dedicated to selling and marketing their products directly influences the capital they are able to generate. As a result, many informal participants were unable to complete the questionnaire on their own. While the researchers assisted by administering the questionnaire verbally, several respondents preferred to focus on engaging passing customers. Among formal retailers, some indicated that they would rather dedicate their time to paid responsibilities, such as addressing customer concerns and managing daily operations. Despite the researchers’ emphasis on participation, several formal retailers declined to participate in the study.

- Uncomfortable to participate

Many informal retailers who declined to participate expressed concerns that participating in the study could expose them to legal risks or jeopardize their businesses. Although the researchers clarified that no personally identifiable information was collected and that the data could not be used against them, these participants still chose not to participate in the research.

- Escort Unavailability (JPC)

The Johannesburg Property Company (JPC) is responsible for registering informal retailers in the city, requiring researchers to obtain permission before data collection. Upon approval, JPC typically provides an escort to accompany the researchers as they engage informal retailers. However, escorts were not always available, which posed challenges, as informal retailers often exhibited hesitation when approached without official accompaniment.

- Lack of incentives

Providing incentives may have motivated retailers to complete the questionnaires, as they would receive an immediate tangible benefit and perceive their time as well-spent.

### 5.3. Interpretation of Neutral Responses in Research Findings

A notable limitation of this study is the interpretation of neutral responses in the Likert-scale questions, particularly regarding awareness of COJMM waste by-laws and the mixing of FVW with other waste types. Despite piloting the questionnaire, such neutral responses may still reflect uncertainty, lack of understanding, reluctance to admit non-compliance, or survey fatigue rather than genuine ambivalence. Future research could consider complementary qualitative approaches to explore the underlying reasons for neutral responses in greater depth.

### 5.4. Limitations: Representativeness and Generalizability

The use of purposive sampling allowed the researchers to target respondents with relevant experience; however, this approach also imposed limitations on representativeness and generalizability. The sample was restricted to a single region of the COJMM and to retailers present during the data collection period. Consequently, the findings cannot be statistically generalized to all retailers in Johannesburg or across South Africa. Certain informal retailers—particularly those who are mobile, absent, or operating informally—may have been underrepresented, while the formal retailer sample was limited to franchise supermarkets, excluding independent grocery stores. As such, the results primarily reflect awareness patterns within the selected retail environments rather than the entire retail sector.

Nonetheless, the study provides meaningful analytical generalizability by highlighting how structural informality, institutional engagement, and corporate governance shape fruit and vegetable waste management in LMIC urban contexts. These insights may be applicable to other metropolitan areas with high levels of informality and waste systems that are predominantly disposal-focused.

## 6. Conclusions

This study investigated FVW governance within the COJMM by assessing awareness levels and management practices of FVW among both formal and informal retailers. The findings highlight that FVW governance in COJMM is characterized by fragmented implementation, limited awareness of municipal waste management by-laws, and a consistent reliance on disposal as a primary practice across both retail sectors. Despite higher awareness and exposure to FVW minimization training among formal retailers, practices such as FVW valorization and donation remained underutilized. Informal retailers displayed significant gaps in awareness and FVW training, accentuating their exclusion from formal waste governance systems. The study contributes to the limited research on FVW governance in the retail sector, particularly within African metropolitan contexts where informal food sectors are fundamental to urban food distribution. The study’s focus on both formal and informal retailers provides a more comprehensive understanding of governance dynamics and underscores the need for inclusive, integrated FVW management strategies. Based on the findings, practical policy implications include strengthening enforcement of municipal waste by-laws, implementing targeted training programs for both formal and informal retailers, integrating informal retailers into municipal waste governance frameworks, and supporting FVW valorization initiatives such as composting, redistribution, and donation schemes.

Future research should expand on these findings by using mixed-methods and longitudinal designs to explore the Behavioral, contractual, and infrastructural factors that influence disposal-centered practices. Studies should also be extended to other metropolitan areas to assess the success of municipal interventions and waste valorization pilots in promoting circular economy strategies. Furthermore, detailed profiles of retail units, including variables such as floor area, years in operation, product range, and turnover, should be included to better understand how structural and operational characteristics impact FVW management across both formal and informal retail settings sectors. Overall, the findings indicate that unsustainable FVW management in COJMM is largely influenced by structural governance issues, including weak enforcement by authorities, limited institutional involvement, and inadequate FVW diversion infrastructure. These insights position COJMM as a microcosm of broader urban FVW governance challenges experienced by LMIC, underscoring the importance of inclusive, comprehensive, and capacity-aware governance approaches that elevate municipal–retailer engagement and promote sustainability through the adoption of FVW prevention strategies and valorization technologies.

## Figures and Tables

**Table 1:** Chi-square test parameters.

Awareness Variable	Demographic Variable	Hypothesis Tested	Purpose
I am aware of the impacts of poor fruit and vegetable waste management. Fruit and vegetable waste is a global concern. I am aware of the COJMM waste management bylaws. Disposal of fruit and vegetable waste is the only effective waste management method.	Age groups	H1a/H1b	Tests whether awareness levels are influenced by age.
	Gender	H2a/H2b	Tests whether awareness levels are influenced by gender.
	Educational level	H3a/H3b	Tests whether awareness levels are influenced by educational level

**Table 2:** Socio-Demographics of Informal Retailers.

Socio-Demographic Factors		Frequency (n)	Percentage (%)
**Gender**	Male	77	65.3
	Female	41	34.7
**Age**	18–29	24	20.3
	30–49	80	67.8
	50–69	13	11.1
	70–89	1	0.8
**Highest educational level**	Never went to school	28	23.7
	Primary	46	39.0
	Secondary	40	33.9
	Tertiary	4	3.4

**Table 3:** Awareness of FVW (informal retailers).

	Agree (%)	Disagree (%)	Neutral (%)	Strongly Agree (%)	Strongly Disagree (%)	Total (%)
I am aware of the impacts of poor fruit and vegetable waste management	39.8	33.1	18.6	1.7	6.8	100.0
Fruit and vegetable waste is a global concern.	28.8	31.4	17.8	0.0	22.0	100.0
I am aware of the City of Johannesburg Metropolitan Municipality waste management by-laws.	11.0	54.2	33.1	1.7	0.0	100.0
Disposal of fruit and vegetable waste is the only effective waste management method.	83.9	1.7	11.0	3.4	0.0	100.0

**Table 4:** Informal retailers’ FVW practices.

	Agree (%)	Disagree (%)	Neutral (%)	Strongly Agree (%)	Strongly Disagree (%)	Total (%)
I am inducted on ways I can use to minimize fruit and vegetable waste	0.0	81.4	13.5	1.7	3.4	100.0
I store my fruit and vegetables in suitable conditions.	32.2	44.9	12.7	10.2	0.0	100.0
Fruit and vegetable waste is mixed with other waste types when disposed of.	42.4	35.6	20.3	1.7	0.0	100.0
I am well inducted on proper fruit and vegetable waste disposal.	28.8	44.9	24.6	1.7	0.0	100.0

**Table 5:** Socio-Demographics of formal Retailers.

Socio-Demographic Factors		Frequency (n)	Percentage (%)
**Gender**	Male	18	41.9
	Female	25	58.1
**Age**	18–29	26	60.5
	30–49	16	37.2
	50–65	1	2.3
**Highest educational level**	Never went to school	0	0
	Primary	0	0
	Secondary	26	60.5
	Tertiary	17	39.5

**Table 6:** Awareness of FVW (formal retailers).

	Strongly Agree (%)	Agree (%)	Neutral (%)	Disagree (%)	Strongly Disagree (%)	Total (%)
I am aware of the impacts of poor fruit and vegetable waste management.	46.5	37.2	16.3	0.0	0.0	100.0
Fruit and vegetable waste is a global concern.	16.2	23.3	14.0	25.6	20.9	100.0
I am aware of the COJMM waste management bylaws	6.9	27.5	54	11.6	0.0	100.0
Disposal of fruit and vegetable waste is the only effective waste management method.	32.6	65.1	2.3	0.0	0.0	100.0

**Table 7:** Formal retailers’ FVW practices.

	Strongly Agree (%)	Agree (%)	Neutral (%)	Disagree (%)	Strongly Disagree (%)	Total (%)
Fruit and vegetable waste is supposed to be mixed with other waste types when disposed of	4.7	0.0	72.1	9.2	14.0	100.0
I store fruit and vegetables in suitable conditions	55.8	44.2	0.0	0.0	0.0	100.0
I am inducted on ways to minimize fruit and vegetable waste	27.9	69.8	0.0	2.3	0.0	100.0

**Table 8:** Chi-square results and hypothesis.

Variable	Questions	*p*-Value	Hypothesis
Age	I am aware of the impacts of poor fruit and vegetable waste management.	<0.001 *	Reject the H1a
	Fruit and vegetable waste is a global concern	<0.001 *	Reject the H1a
	I am aware of the COJMM waste management bylaws.	0.030 *	Reject the H1a
	Disposal of fruit and vegetable waste is the only effective waste management method.	0.802	Accept the H1a
Gender	I am aware of the impacts of poor fruit and vegetable waste management.	<0.001 *	Reject the H2a
	Fruit and vegetable waste is a global concern	0.001 *	Reject the H2a
	I am aware of the COJMM waste management bylaws.	0.020 *	Reject the H2a
	Disposal of fruit and vegetable waste is the only effective waste management method.	0.005 *	Reject the H2a
Educational Level	I am aware of the impacts of poor fruit and vegetable waste management.	<0.001 *	Reject the H3a
	Fruit and vegetable waste is a global concern	<0.001 *	Reject the H3a
	I am aware of the COJMM waste management bylaws.	<0.001 *	Reject the H3a
	Disposal of fruit and vegetable waste is the only effective waste management method.	0.092	Accept the H3a

* *p* < 0.05 Statistically significant.

**Table 9:** Chi-square results and hypothesis.

Variable	Questions	*p*-Value	Hypothesis
Age	I am aware of the impacts of poor fruit and vegetable waste management.	0.158	Accept the H1a
	Fruit and vegetable waste is a global concern	0.173	Accept the H1a
	I am aware of the COJMM waste management bylaws.	0.069	Accept the H1a
	Disposal of fruit and vegetable waste is the only effective waste management method.	0.005 *	Reject the H1a
Gender	I am aware of the impacts of poor fruit and vegetable waste management.	0.155	Accept the H2a
	Fruit and vegetable waste is a global concern	0.813	Accept the H2a
	I am aware of the COJMM waste management bylaws.	0.738	Accept the H2a
	Disposal of fruit and vegetable waste is the only effective waste management method.	0.805	Accept the H2a
Educational level	I am aware of the impacts of poor fruit and vegetable waste management.	0.504	Accept the H3a
	Fruit and vegetable waste is a global concern	0.775	Accept the H3a
	I am aware of the COJMM waste management bylaws.	0.637	Accept the H3a
	Disposal of fruit and vegetable waste is the only effective waste management method.	0.480	Accept the H3a

* *p* < 0.05 Statistically significant.

## Data Availability

The original contributions presented in this study are included in the article. Further inquiries can be directed to the corresponding authors.

## Abbreviations

The following abbreviations are used in this manuscript:

LMIC	Low- and Middle-Income Countries
FVW	Fruit and Vegetable Waste
FV	Fruit and Vegetables
COJMM	City of Johannesburg Metropolitan Municipality
CBD	Central Business District
